# The proteasome deubiquitinase inhibitor b-AP15 enhances DR5 activation-induced apoptosis through stabilizing DR5

**DOI:** 10.1038/s41598-017-08424-w

**Published:** 2017-08-14

**Authors:** You-Take Oh, Liang Deng, Jiusheng Deng, Shi-Yong Sun

**Affiliations:** Department of Hematology and Medical Oncology, Winship Cancer Institute, Emory University School of Medicine, Atlanta, Georgia USA

## Abstract

b-AP15 and its derivatives block proteasome deubiquitinase (DUB) activity and have been developed and tested in the clinic as potential cancer therapeutic agents. b-AP15 induces apoptosis in cancer cells, but the underlying mechanisms are largely undefined. The current study focuses on studying the modulatory effects of b-AP15 on death receptor 5 (DR5) levels and DR5 activation-induced apoptosis as well as on understanding the underlying mechanisms. Treatment with b-AP15 potently increased DR5 levels including cell surface DR5 in different cancer cell lines with limited or no effects on the levels of other related proteins including DR4, c-FLIP, FADD, and caspase-8. b-AP15 substantially slowed the degradation of DR5, suggesting that it stabilizes DR5. Moreover, b-AP15 effectively augmented apoptosis when combined with TRAIL or the DR5 agonistic antibody AMG655; these effects are DR5-dependent because DR5 deficiency abolished the ability of b-AP15 to enhance TRAIL- or AMG655-induced apoptosis. Therefore, it is clear that b-AP15, and possibly its derivatives, can stabilize DR5 and increase functional cell surface DR5 levels, resulting in enhancement of DR5 activation-induced apoptosis. Our findings suggest that b-AP15 and its derivatives may have potential in sensitizing cancer cells to DR5 activation-based cancer therapy.

## Introduction

Targeting the ubiquitin-proteasome system (UPS), a conserved pathway in the regulation of some critical biological processes such as protein turnover, has emerged as a promising strategy for the development of novel anti-cancer therapies since cancer cells are assumed to be dependent on a functional UPS^1^. Ubiquitinated proteins are degraded by the 26S proteasome, which comprises a proteolytic 20S core particle capped by 19S regulatory particles. Beyond the proteasome inhibitors bortezomib (BTZ; also called PS-341) and carfilzomib (CFZ), which are FDA-approved anticancer drugs that target the 20S core, another group of small molecules including b-AP15 and its derivatives that block the deubiquitinase (DUB) activity of the 19S regulatory particle without inhibiting the proteolytic activity of the 20S core particle have been developed and tested in the clinic as potential cancer therapeutic agents^1–3^. b-AP15 inhibits two 19S regulatory particle-associated DUBs, USP14 and UCHL5, resulting in the rapid accumulation of high molecular weight ubiquitin conjugates and functional proteasome shutdown, as is caused by proteasome inhibitors^1^. Several studies have shown that b-AP15 induces apoptosis of cancer cells, which serves as its major anticancer mechanism^2, 4–7^. Induction of oxidative stress and ER stress has been suggested to account for b-AP15-induced apoptosis^4^. Otherwise, the mechanisms by which b-AP15 induces apoptosis of cancer cells are largely unclear.

Death receptor 5 (DR5; also known as TRAIL-R2) is located at the cell surface and becomes activated upon binding to its ligand tumor necrosis factor-related apoptosis inducing ligand (TRAIL) or being aggregated induced by an agonistic antibody. Activated DR5 initiates apoptosis through Fas-associated death domain (FADD)-dependent recruitment and activation of caspase-8 and eventual caspase 8-mediated activation of caspase cascades. This process is inhibited by cellular FLICE-inhibitory protein (c-FLIP) through competing with caspase-8 to bind to FADD at the death-inducing signaling complex (DISC), blocking caspase-8 activation and final apoptosis^8, 9^. Given that TRAIL is endogenously produced by several types of immune cells such as cytotoxic T cells and natural killer (NK) cells^10^, the induction of apoptosis by ligation of endogenous TRAIL with DR5 is a critical mechanism underlying the immune surveillance of cancer cells^10, 11^. Moreover, soluble recombinant human TRAIL and DR5 agonistic antibodies that activate DR5-dependent apoptosis are also potential anticancer therapeutics^8, 12–14^.

DR5, its sibling death receptor 4 (DR4), and other DISC proteins including FADD, caspase-8, and c-FLIP are known to be regulated by the ubiquitin-proteasome system. The E-3 ligase c-Cbl binds to both DR5 and DR4 and induces their monoubiquitination, resulting in internalization and degradation^15^. Accordingly, knockdown of c-Cbl increases the levels of DR5 and DR4, leading to sensitization of TRAIL-induced apoptosis^16^. A recent study has shown that the membrane-associated RING-CH-8 (MARCH-8) ligase interacts with and ubiquitinates DR4, facilitating its internalization and degradation^17^. Makorin ring finger protein 1 (MKRN1) E3 ligase has been shown to mediate ubiquitination and proteasomal degradation of FADD. MKRN1 knockdown results in FADD protein stabilization and rapid formation of the DISC and sensitization to extrinsic apoptosis^18^. The polyubiquitination of caspase-8 is positively regulated by a cullin3 (CUL3)-based E3 ligase through the RING box protein RBX1, and can be reversed by the deubiquitinase A20^19^. c-FLIP has long been recognized as an unstable protein undergoing ubiquitination and proteasome degradation^20–22^.

A previous study showed that b-AP15 elevated cell surface DR5 accompanied with reduction of c-FLIP in some cancer cell lines and enhanced killing of cancer cells by natural killer cells and T cells through TRAIL-induced apoptosis^23^. However the underlying mechanism by which b-AP15 elevates DR5 levels has not been elucidated. The different UAB inhibitor PR-619 was shown to increase caspase-8 ubiquitination and caspase-8 enzymatic activity in normal human fibroblasts and eventually sensitize these cells to TRAIL-mediated apoptosis^24^. Our recent study has shown that the proteasome inhibitor, CFZ, elevates DR5 levels through protein stabilization, induces DR5-dependent apoptosis and enhances TRAIL-induced apoptosis^25^. The current study thus focuses on demonstrating the modulatory effects of b-AP15 on DR5 and other DISC components, and the impact on DR5 activation-induced apoptosis and underlying mechanisms.

## Results

### b-AP15 enhances the levels of DR5, but not other DISC components, in human cancer cells

**Figure 1:**
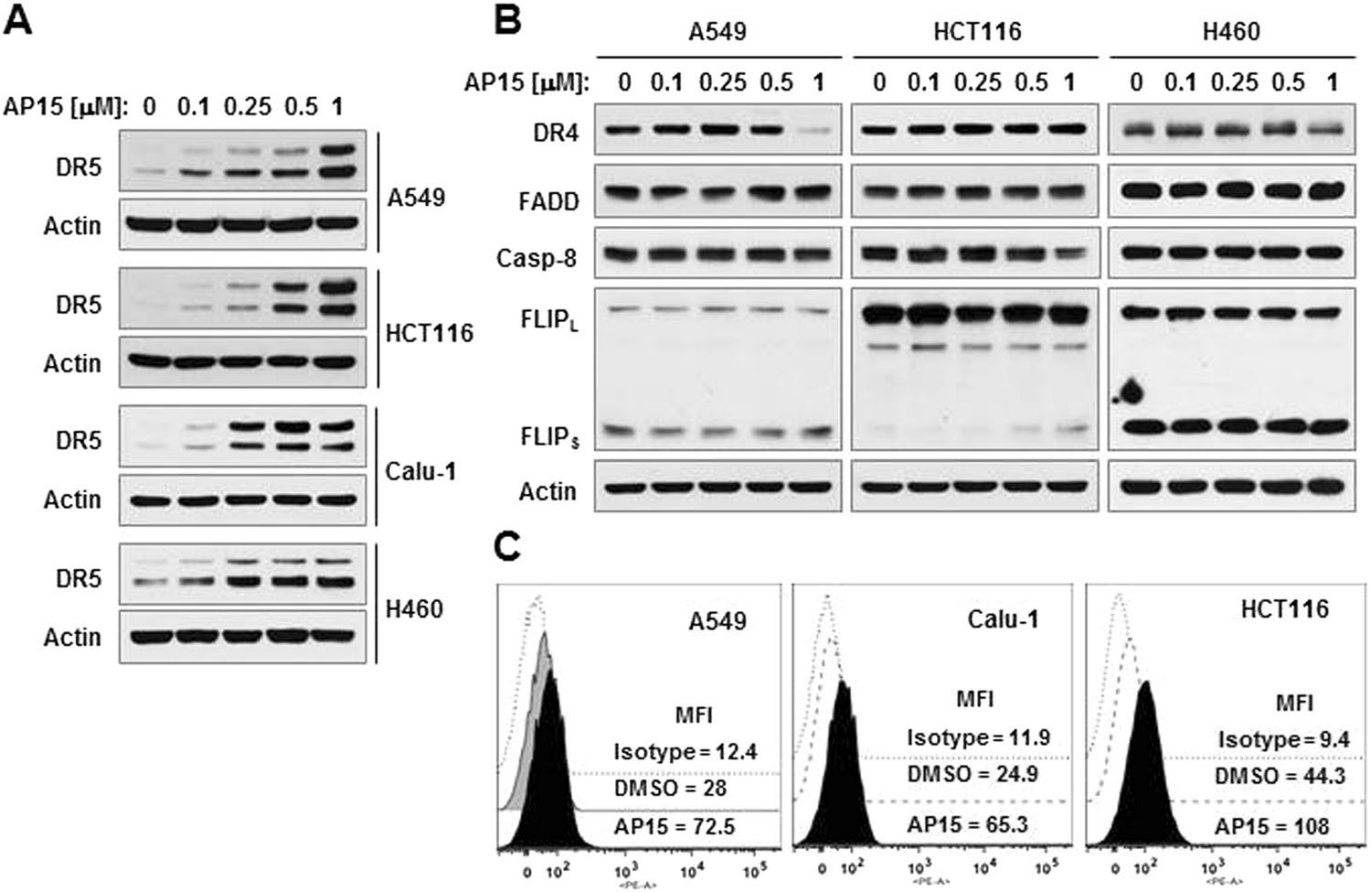
b-AP15 primarily increases the levels of DR5 (**A** and **C**), but not other DISC components (**B**), in cancer cells. (**A** and **B**) The indicated cancer cell lines were exposed to varied concentrations of b-AP15 as shown for 9 h and then harvested for preparation of whole-cell protein lysates and subsequent Western blotting. Full-length blots/gels are presented in Supplementary Figure S1. (**C**) The given cell lines were treated with 0.5 μM b-AP15 for 8 h and then harvested for staining of DRs followed by flow cytometric analysis of cell surface DR5. The dashed-line open peaks represent DMSO-treated cells stained with a matched control PE-conjugated IgG isotype antibody. The filled gray or dotted-line open peaks are DMSO-treated cells stained with PE-conjugated anti-DR5 antibody. The filled black peaks show b-AP15 treated cells stained with PE-conjugated anti-DR5 antibody. MFI for each sample was indicated.

The DISC components, DR5, DR4, c-FLIP, caspase-8, and FADD, are known to be regulated by protein ubiquitination^15, 17–20, 22^. We thus determined whether b-AP15 modulates the levels of these proteins. As presented in Fig. 1, b-AP15 at concentration ranges from 0.1 to 1 μM increased DR5 levels in every tested cancer cell line including A549, HCT116, Calu1, and H460 in a concentration-dependent manner (Fig. 1A). However, under the same conditions, b-AP15 did not change the levels of FADD, FLIP, or caspase-8. At a certain range of concentrations, b-AP15 minimally increased DR4 levels in some cell lines (A549 and HCT116) (Fig. 1B). Moreover, b-AP15 effectively increased the levels of cell surface DR5 in the 3 tested cell lines (Fig. 1C). These results demonstrate that b-AP15 primarily elevates DR5 levels including the levels of functional cell surface DR5.

### b-AP15 stabilizes DR5 protein

**Figure 2:**
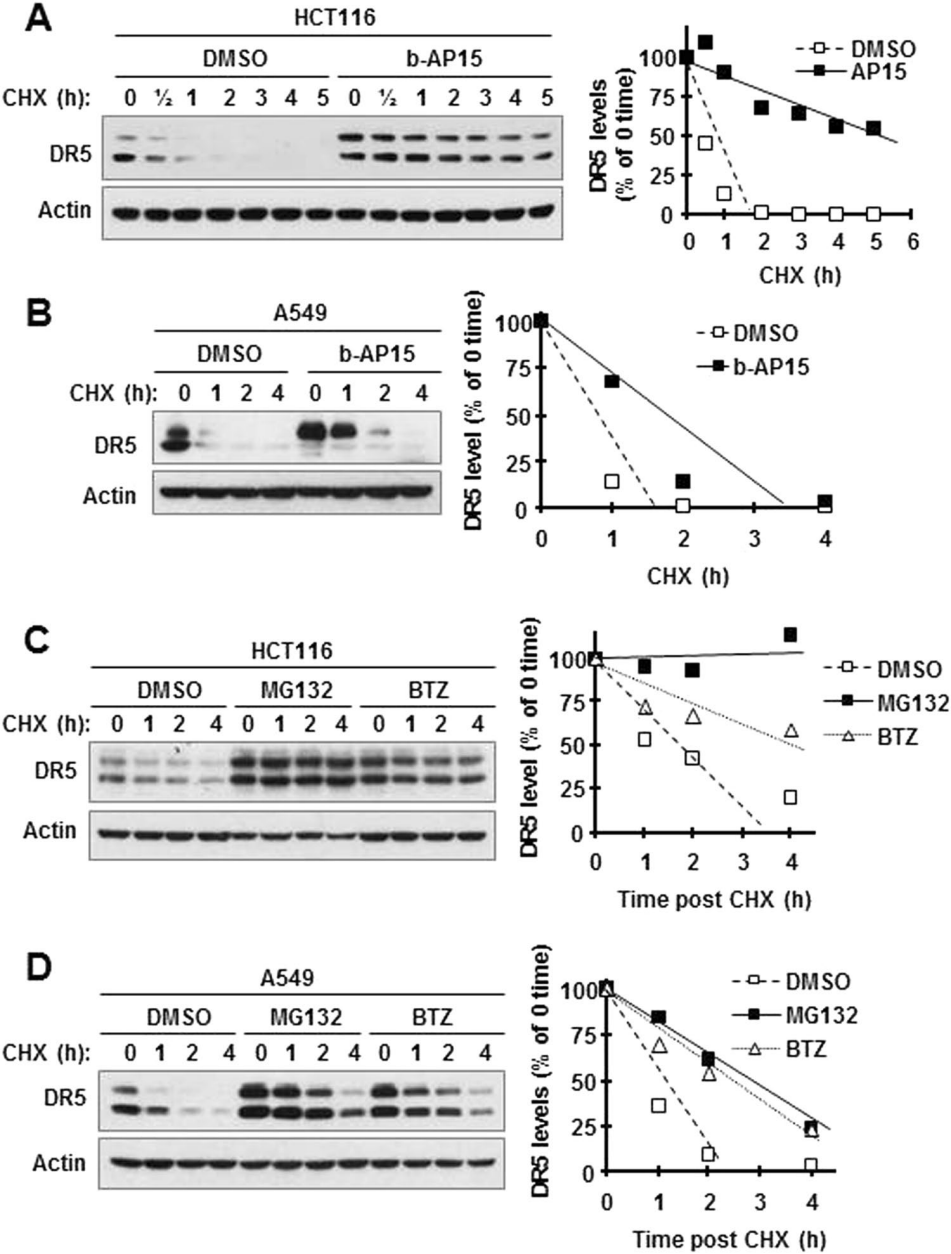
b-AP15 (**A** and **B**) and proteasome inhibitors (**C** and **D**) suppress DR5 degradation. Then indicated cancer cell lines were treated with DMSO or 0.5 μM b-AP15 for 8 h (**A** and **B**) or with DMSO, 10 μM MG132 or 100 nM BTZ for 5 h (**C** and **D**) followed by the addition of 10 μg/ml CHX. At different times as indicated post CHX, the cells were harvested for preparation of whole-cell protein lysates and subsequent Western blotting. Protein levels were quantified with NIH Image J software (Bethesda, MA) and were normalized to actin. The results were plotted as the relative DR5 levels compared to those at the time 0 of CHX treatment (right panel). Full-length blots/gels are presented in Supplementary Figure S2.

To determine the mechanism by which b-AP15 increases DR5 levels, we examined the effect of b-AP15 on modulation of DR5 degradation or stability. By conducting a cycloheximide (CHX) chase assay, we found that the rate of DR5 degradation in b-AP15-treated cells was much slower than in DMSO control cells in both HCT116 and A549 cells (Fig. 2A and B), indicating that b-AP15 suppresses DR5 degradation or stabilizes DR5 protein. Given that b-AP15 is a proteasome DUB inhibitor, we further examined the effects of the proteasome inhibitors, MG132 and BTZ, on DR5 degradation. In a similar fashion as b-AP15 did, both MG132 and BTZ, apparently slowed down the degradation of DR5 in both HCT116 and A539 cells, indicating stabilization of DR5 (Fig. 2C and D).

### b-AP15 enhances TRAIL-induced apoptosis

**Figure 3:**
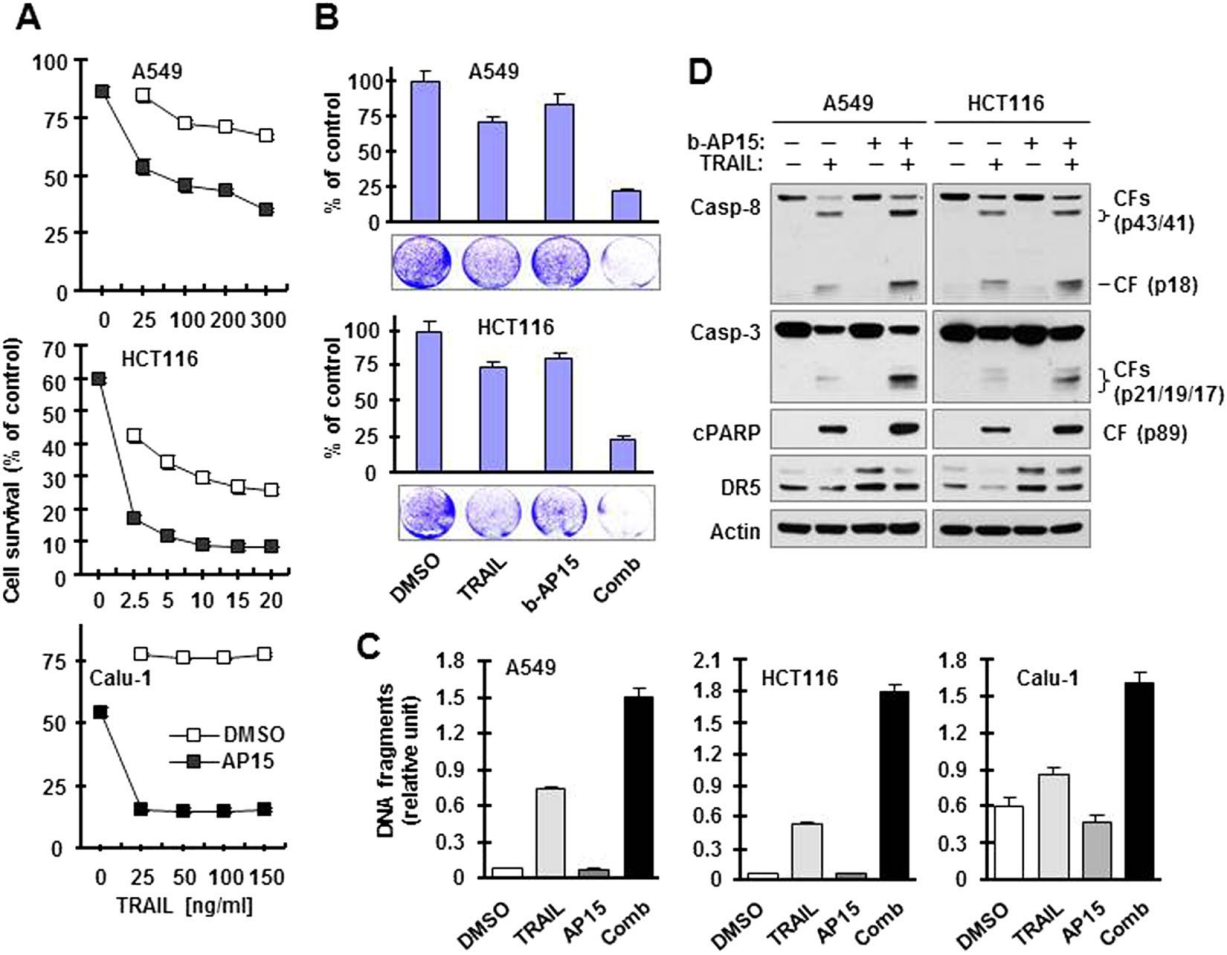
b-AP15 enhances the effects of TRAIL in decreasing cell survival (**A** and **B**) and inducing apoptosis (**C** and **D**) in cancer cells. (**A**) Different cancer cell lines in 96-well plates were exposed to DMSO or 0.5 μM b-AP15 for 3 h followed by co-treatment with varied concentrations of TRAIL for an additional 24 h. Cell numbers were the estimated with the SRB assay. The data are means ± SDs of four replicate determinations. (**B**) The given cell lines in 12-well plates were pre-treated with DMSO or 0.1 μM b-AP15 for 4 h and then co-treated with 50 ng/ml (A549) or 5 ng/ml (HCT116) TRAIL for an additional 60 h. The cells were then stained with crystal violet dye. The data are means ± SDs of triplicate determinations. (**C** and **D**) The indicated cell lines were exposed to 0.5 μM b-AP15 for 3 h followed by co-treatment with 100 ng/ml (A549 and Calu-1) or 10 ng/ml (HCT116) TRAIL for an additional 4 h. The cells were then harvested to measure DNA fragments with a cell death detection ELISA kit (**C**) and to detect the cleavage of caspases and PARP (**D**). The data are means ± SDs of triplicate determinations. CF, cleaved fragment. Full-length blots/gels are presented in Supplementary Figure S3.

Since b-AP15 increases the levels of DR5 including cell surface DR5, we then determined whether b-AP15 sensitizes cancer cells to TRAIL-induced apoptosis. We first examined the impact of b-AP15 on the survival of cancer cells in a 24 h assay and found that the presence of b-AP15 effectively enhanced the killing effects of TRAIL in every tested cell line (Fig. 3A). In a long-term (60 h) survival assay, the combination of b-AP15 and TRAIL was significantly more active than either agent alone in decreasing the survival of the tested cell lines (Fig. 3B). In agreement, the combination of b-AP15 and TRAIL was also significantly more potent than each single agent in increasing DNA fragmentation (Fig. 3C) and inducing cleavage of caspase-8, caspase-3 and PARP (Fig. 3D). These results together clearly show that b-AP15 significantly sensitizes cancer cells to TRAIL-mediated killing, likely though effectively enhancing TRAIL-induced apoptosis.

### b-AP15 augments apoptosis induced by the DR5 agonistic antibody AMG655

**Figure 4:**
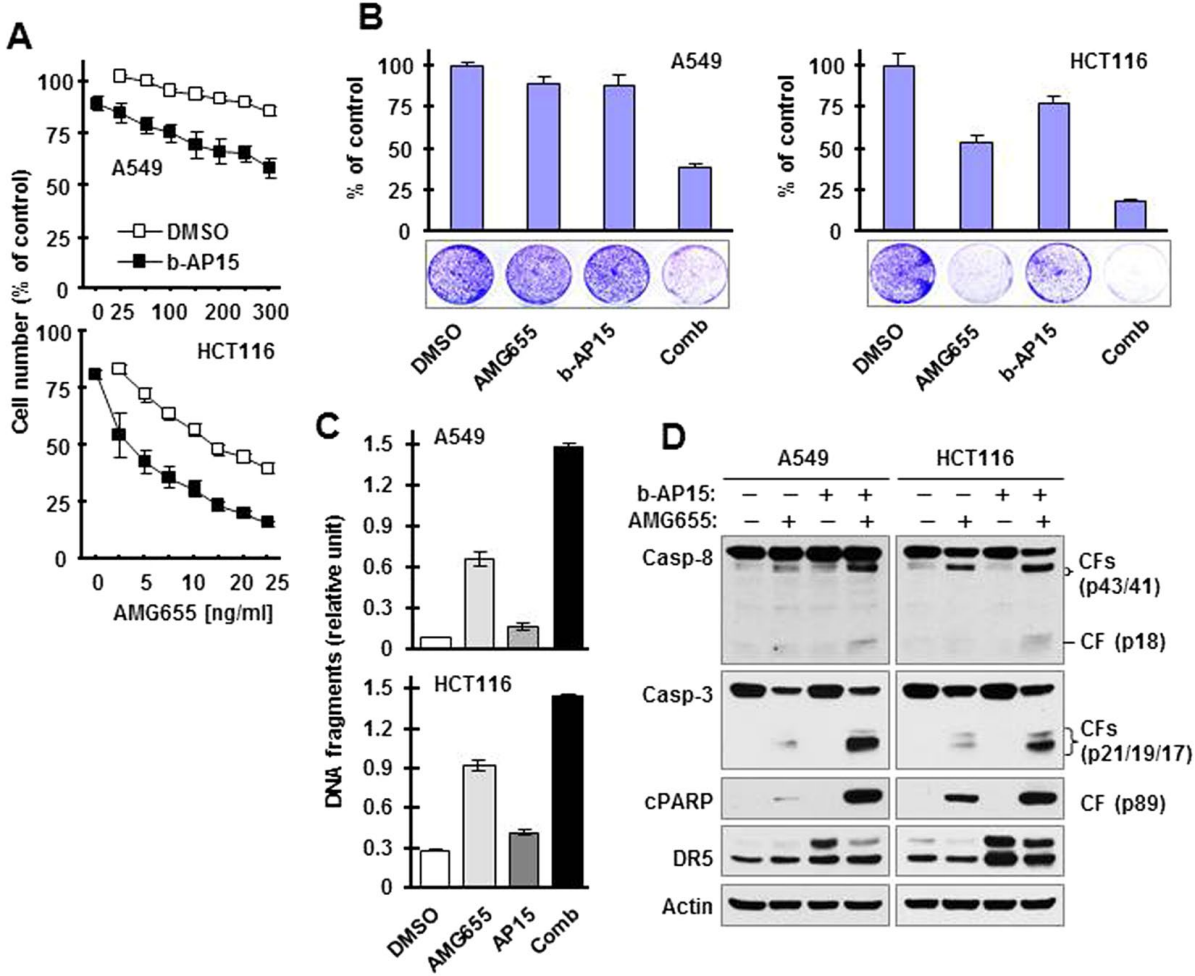
b-AP15 enhances the effects of AMG655 in decreasing cell survival (**A** and **B**) and inducing apoptosis (**C** and **D**) in cancer cells. (**A**) Different cancer cell lines in 96-well plates were exposed to DMSO or 0.5 μM b-AP15 for 3 h followed by co-treatment with varied concentrations of AMG655 for an additional 24 h. Cell numbers were the estimated with the SRB assay. The data are means ± SDs of four replicate determinations. (**B**) The given cell lines in 12-well plates were pre-treated with DMSO or 0.1 μM b-AP15 for 4 h and then co-treated with 100 ng/ml (A549) or 10 ng/ml (HCT116) AMG655 for an additional 60 h. The cells were then stained with crystal violet dye. The data are means ± SDs of triplicate determinations. (**C**) and (**D**) The indicated cell lines were exposed to 0.5 μM b-AP15 for 3 h followed by co-treatment with 100 ng/ml (A549) or 10 ng/ml (HCT116) AMG655 for an additional 4 h. The cells were then harvested to measure DNA fragments with a cell death detection ELISA kit (**C**) and to detect the cleavage of caspases and PARP (**D**). The data are means ± SDs of triplicate determinations. CF, cleaved fragment. Full-length blots/gels are presented in Supplementary Figure S4.

With the same rationale as for TRAIL, we speculated that b-AP15 might sensitize cancer cells to apoptosis induced by AMG655, a DR5 agonistic antibody that triggers apoptosis through inducing cell surface DR5 aggregation^26^. Similar to our observations with TRAIL, b-AP15 augmented the effects of AMG655 in decreasing cell survival (Fig. 4A and B), increasing DNA fragmentation (Fig. 4C), and enhancing cleavage of caspase-8, caspase-3 and PARP (Fig. 4D) in both A549 and HCT116 cells. Hence b-AP15 also augments AMG655-induced apoptosis.

### b-AP15 enhancement of apoptosis by TRAIL or AMG655 requires DR5 elevation

**Figure 5:**
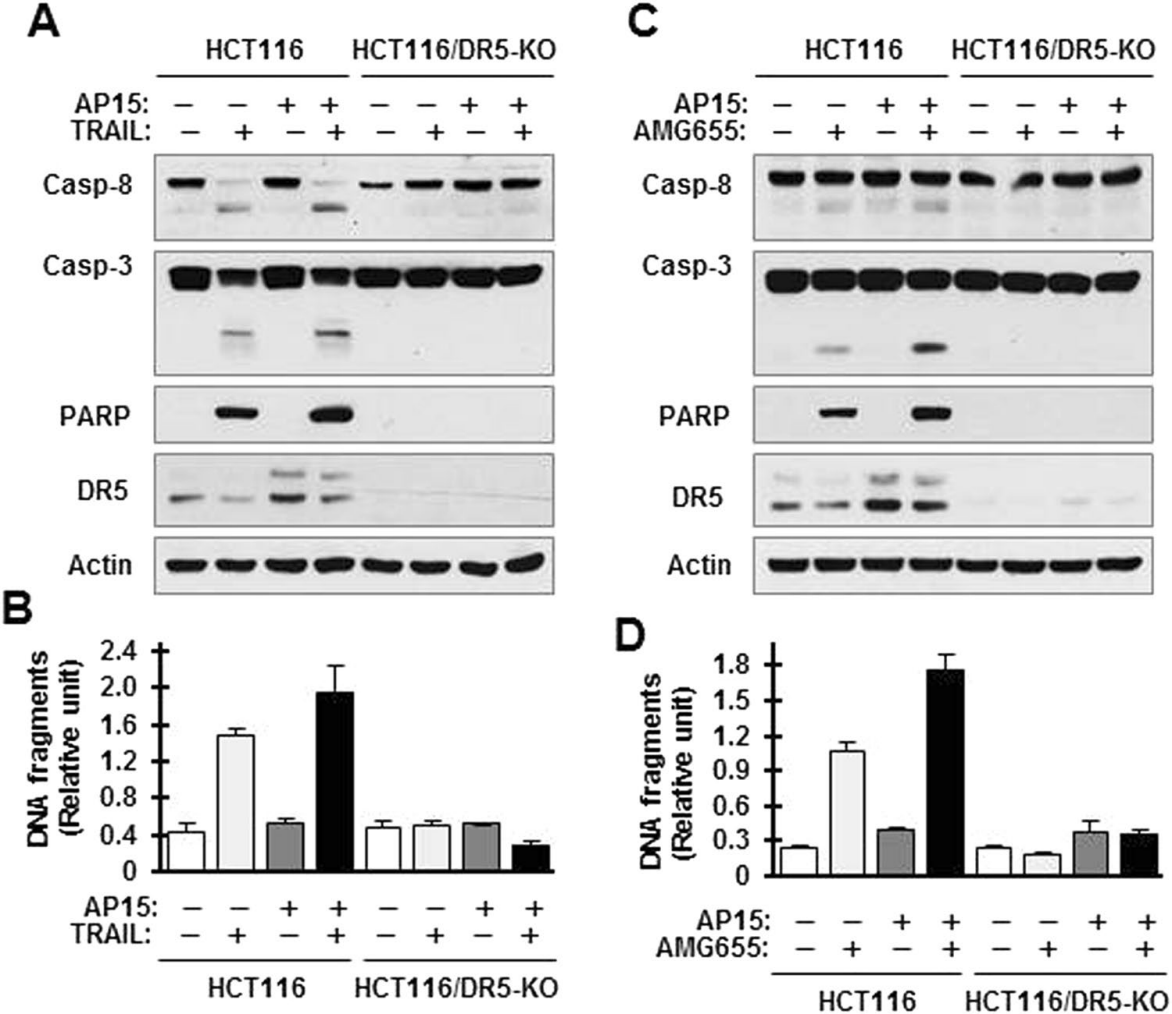
DR5 deficiency abolishes the enhanced induction of apoptosis by the combination of b-AP15 with TRAIL (**A** and **B**) or AMG655 (**C** and **D**). The indicated cell lines were pretreated with 0.5 μM b-AP15 for 4 h and then co-treated with 20 ng/ml TRAIL (**A** and **B**) or 40 ng/ml AMG655 (**C** and **D**) for an additional 4 h. The cells were then harvested to measure DNA with a cell death ELISA kit (**B** and **D**) and to detect the cleavage of caspases and PARP with Western blotting (**A** and **C**). The data are means ± SDs of triplicate determinations. CF, cleaved fragment. Full-length blots/gels are presented in Supplementary Figure S5.

To demonstrate the critical role of DR5 elevation in mediating the enhancement of TRAIL- or AMG655-induced apoptosis by b-AP15, we compared the enhancement of TRAIL- and AMG655-induced apoptosis by b-AP15 in HCT116 and HCT116/DR5-KO cells. The combination of b-AP15 and TRAIL (Fig. 5A and B) or AMG655 (Fig. 5C and D) enhanced DNA fragmentation (Fig. 5B and D) and cleavage of caspase-8, caspase-3, and PARP (Fig. 5A and C) in HCT116 cells as demonstrated above, but failed to do so in HCT116/DR5-KO cells. Therefore it is clear that loss of DR5 fully protects cancer cells from undergoing apoptosis enhanced by b-AP15 combined with TRAIL or AMG655, indicating an essential role of DR5 elevation in the enhancement of TRAIL- or AMG655-induced apoptosis by b-AP15.

### b-AP15 functions similarly to proteasome inhibitors in elevating DR5 levels and enhancing TRAIL-induced apoptosis

**Figure 6:**
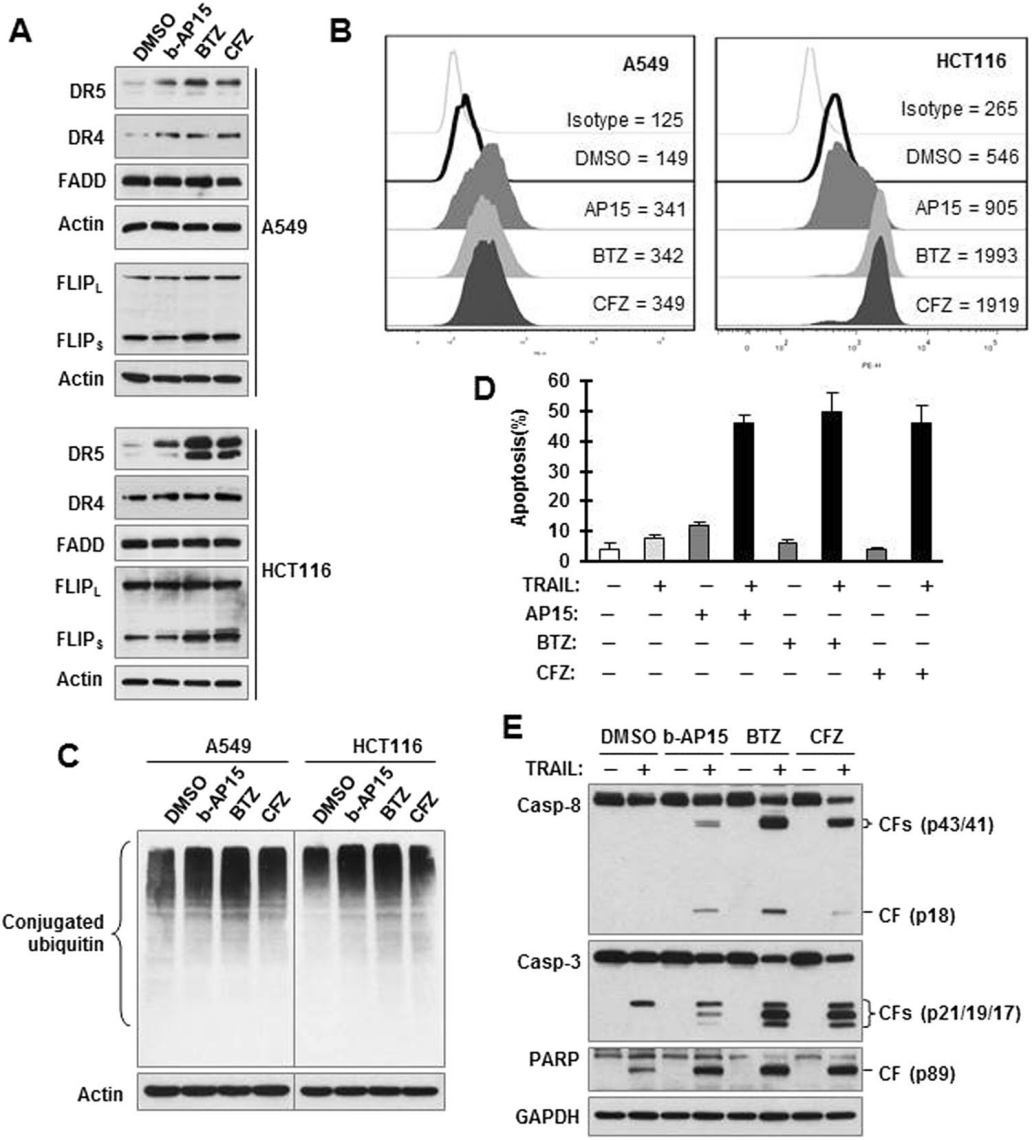
b-AP15 functions similarly to proteasome inhibitors in elevating DR5 levels (**A** and **B**), accumulating conjugated ubiquitin (**C**) and enhancing TRAIL-induced apoptosis (**D** and **E**). (**A–C**) The indicated cell lines were exposed to 0.5 μM b-AP15, 0.1 μM BTZ, or 0.1 μM CFZ for 8 h (**A** and **C**) or 10 h (**B**) and then harvested for detection of different proteins and protein polyubiquitination with Western blotting (**A** and **C**) or for detection of cell surface DR5 with flow cytometry (**B**). The numbers inside (**B**) are MFIs of DR5 staining. (**D** and **E**) A549 cells were exposed to 0.5 μM b-AP15, 0.1 μM BTZ, or 0.1 μM CFZ alone, 50 ng/ml TRAIL alone, or their respective combinations for 10 h or 20 h and then harvested to detect apoptosis with annexin V staining/flow cytometry (**D**) and to detect the cleavage of caspases and PARP with Western blotting (**E**). The data in (**D**) are means ± SDs of triplicate determinations. CF, cleaved fragment. Full-length blots/gels are presented in Supplementary Figure S6.

We compared the effects of b-AP15 with those of the proteasome inhibitors, CFZ and BTZ, on DR5 levels and TRAIL-induced apoptosis. Treatment with b-AP15, CFZ and BTZ all increased DR5 levels including levels of cell surface DR5 with limited or no elevation of DR4 and FADD levels (Fig. 6A and B). However, CFZ and BTZ, but not b-AP15, elevated FLIP_S_ levels as we previously reported^25, 27^. Under the tested conditions, treatment with b-AP15 increased the amounts of conjugated ubiquitin or protein polyubiquitination, as did CFZ and BTZ (Fig. 6C). Moreover, b-AP15, CFZ and BTZ also augmented TRAIL-induced apoptosis, as evidenced by the detection of greater numbers of annexin V-positive cells and higher levels of cleaved caspase-8, caspase-3, and PARP in cells exposed to TRAIL combined with any of the inhibitors than in cells exposed to TRAIL alone or a given inhibitor alone (Fig. 6D and E).

### b-AP15 function differently from lysosome inhibitors in modulating total and cell surface DR5 levels and TRAIL-induced apoptosis

**Figure 7:**
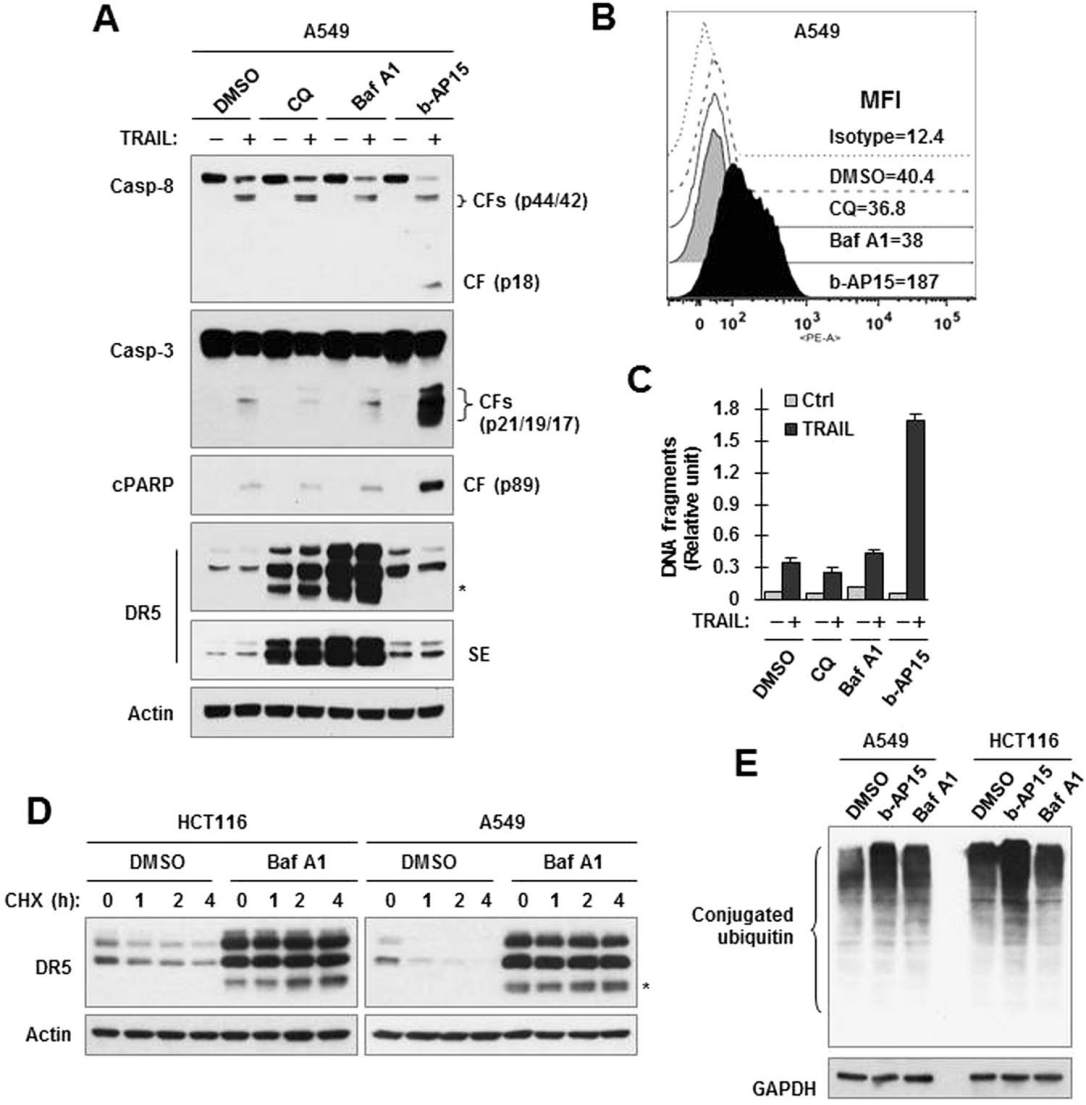
Inhibition of lysosome with both CQ and Baf A1 does not increase cell surface DR5 levels and protein polyubiquitinaiton (**B** and **D**) and enhances TRAIL-induced apoptosis (**A** and **C**) albeit potently elevating total cellular levels of DR5 (**C**) and increasing DR5 stability (**D**). (**A** and **C**) A549 cells were exposed to 20 μM CQ, 50 nM Baf A or 1 μM b-AP15 for 8 h and then co-treated with 200 ng/ml TRAIL for additional 4 h. (**B**) A549 cells were exposed to 20 μM CQ, 50 nM Baf A or 1 μM b-AP15 for 8 h. (**D)** Both A549 and HCT116 cell lines were treated with DMSO or 50 nM Baf A1 for 5 h followed by the addition of 10 μg/ml CHX. (**E**) Both A549 and HCT116 cell lines were exposed to DMSO, 1 μM b-AP15 or 50 nM Baf A1 for 8 h. After the aforementioned treatments, the cells were harvested for preparation of whole-cell protein lysates and subsequent Western blotting (**A**,**D** and **E**), for cell surface DR5 detection with flow cytometry (**B**) or for detection of apoptosis with the Cell Death Detection ELISA Plus kit (**C**). The data are means ± SDs of triplicate determinations (**C**). SE, short exposure. The asterisk indicates an additional band lower than 40 kD. Full-length blots/gels are presented in Supplementary Figure S7.

Some lysosomal inhibitors such as chloroquine (CQ) were reported to elevate DR5 levels^28^. We noticed that the two lysosomal inhibitors, CQ and bafilomycin A1 (Baf A1), strongly elevated the levels of total cellular DR5 with induction of an additional band lower than 40 kD, which was not induced by b-AP15 (Fig. 7A). However, they did not increase cell surface amounts of DR5 while b-AP15 potently increased cell surface levels of DR5 (Fig. 7B). Moreover, b-AP15, but not CQ and Baf A, enhanced the ability of TRAIL to induce cleavage of caspases (Fig. 7A) and to increase DNA fragmentation (Fig. 7C) even though both CQ and Baf A1 were much more potent than c-AP15 in elevating total cellular levels of DR5 (Fig. 7A). These findings clearly indicate that b-AP15 and lysosomal inhibitors exert different effects on modulating total and surface levels of DR5 and on TRAIL-induced apoptosis. We further examine the effects of Baf A1 on DR5 degradation in the CHX chase assay and observed that Baf A1 suppressed DR5 degradation in both HCT116 and A549 cells (Fig. 7D). Again we noted that Baf A1 induced appearance of an additional band of DR5 smaller than 40 kD in these two cell lines (Fig. 7D). Finally we compared b-AP15 with Baf A1 on protein polyubiquitination and found that b-AP15, but not Baf A1, increased the levels of conjugated ubiquitin or protein polyubiquitination (Fig. 7E).

## Discussion

Ubiquitinated proteins are degraded by the 26S proteasome, which comprises a proteolytic 20S core particle capped by 19S regulatory particles. Inhibition of the proteasome with either the conventional proteasome inhibitors such as BTZ and CFZ that target the 20S core particle or DUB inhibitors such as b-AP15 that abrogate DUB activity of the 19S regulatory particle eventually results in the accumulation of conjugated ubiquitin or ubiqutinylated proteins^1, 2^. Indeed, treatment with b-AP15 elevated the levels of conjugated ubiquitin or polyubiquitinated proteins in our tested cell lines, as did BTZ and CFZ (Fig. 6C), thus validating its targeting of the ubiquitin-proteasome process leading to accumulation of polyubiquitinated proteins in our cell systems.

DR5, DR4, and other DISC components including FADD, caspase-8, and c-FLIP are known to be regulated by proteasome/ubiquitination mechanisms^15–20^. However, the current study has demonstrated that b-AP15 primarily elevates the levels of DR5 but not DR4, FADD, caspase-8, or c-FLIP. Moreover, b-AP15 slowed the degradation of DR5 as demonstrated in both HCT116 and A549 cells. Hence it is clear that b-AP15 increases DR5 levels through stabilization of DR5 protein. This effect is similar to our observations with CFZ, which also stabilizes DR5 protein in the same cell systems^25^ and as demonstrated in the current study (Fig. 2C and D). In contrast to proteasome inhibitors such as CFZ and BTZ that potently elevate the levels of FLIP_S_ while increasing DR5 levels as we reported previously^25, 27^ and confirmed in this study (Fig. 6A), b-AP15 did not increase FLIP_S_ levels. Similar to BTZ and CFZ^25, 27^, b-AP15 effectively increased cell surface DR5 levels and enhanced apoptosis induced by both TRAIL and AMG655, a DR5 agonistic antibody, in different cancer cell lines in a DR5-dependent manner, as evidenced by DR5 deficiency fully protecting cancer cells from undergoing apoptosis when treated with b-AP15 in combination with either TRAIL or AMG655 (Fig. 5). Therefore, it is clear that b-AP15 shares some similar functions (e.g., DR5 elevation and enhancement of DR5 activation-induced apoptosis) with proteasome inhibitors, but lacks some unneeded functions (e.g., FLIP_S_ elevation) that proteasome inhibitors possess. c-FLIP is a key inhibitor of DR5 activation-induced apoptosis^29^. Sustained elevation of FLIP_S_ may in turn attenuate TRAIL- or DR5 activation-induced apoptosis. Our previous finding showed that c-FLIP knockdown further enhanced BTZ-induced apoptosis^27^. From this perspective, b-AP15 has an advantage over proteasome inhibitors regarding modulation of c-FLIP.

In addition to the elevation of cell surface DR5, a previous study reported that b-AP15 reduced c-FLIP levels in some cancer cell lines^23^. However we did not find that b-AP15 altered the levels of either FLIP_L_ or FLIP_S_ in cancer lines we tested (Figs 1B and 6A). The discrepancy between these two studies may be due to the different cancer cell lines used. Based on our extensive research experience on c-FLIP, it is also very likely that the different results were generated from the use of different antibodies in the previous study and our current report. The antibody against c-FLIP used in our study has been validated in many of our previous studies^21, 27, 30–37^.

CQ was recently reported to elevate DR5 levels and enhance TRAIL-induced apoptosis in human renal cancer Caki cells^28^. However we failed to demonstrate the ability of both CQ and Baf A1 in increasing cell surface of DR5 and enhancing TRAIL-induced apoptosis in our lung cancer A549 cells although they elevated total cellular levels of DR5 and stabilized DR5 (Fig. 7). In agreement with our findings, another recent study also reported that CQ induced cytoplasmic localization of DR5 and did not modulate TRAIL-induced apoptosis in human colon cancer HCT116 cells^38^. Moreover lysosome inhibition induced an additional isoform of DR5 protein smaller than 40 kD (Fig. 7), which was not induced by either b-AP15 or proteasome inhibitors tested in this study. Moreover, b-AP15 also shows difference from Baf A1 in affecting protein polyubiquitination because b-AP51, but not Bal A1, elevated ubiquitinated protiens (Fig. 7). Therefore it is apparent that b-AP15 functions differently from lysosome inhibitors in modulating DR5 levels and TRAIL-induced apoptosis. Further study on the mechanism underlying induction of this additional isoform of DR5 by lysosome inhibition is needed and will be our planning direction.

DR5 de novo synthesis and post translational modification such as ubiquitination and degradation, like other proteins, occurs in cytoplasm and functions at cell surface as a membrane protein. As other membrane proteins do, DR5 also undergoes internalization and endocytosis^39^. It is recognized that, after endocytosis, some membrane proteins return or recycle to the cell surface, while some are delivered to late endosome and eventually progressed to lysosome, where they are degraded and cannot go back to cell membrane^40^. Given the fact that lysosome inhibition with both CQ and Baf A1 failed to increase cell surface DR5 levels although stabilizing and increasing total cellular DR5 amounts, we favor that b-AP15, like other proteasome inhibitors do, elevates DR5 levels including cell surface DR5 through stabilizing de novo DR5 by suppressing its proteasomal degradation. This is in agreement with its function feature as a proteasome DUB inhibitor. It has been shown that lysosomal inhibition (e.g., with Baf A1) fails to elevate the levels of ubiquitinated proteins unless proteasome is inhibited^41^. The fact that b-AP15, but not Baf A1, is able to elevate ubuiquitinated protein levels (Fig. 7) further supports the function of b-AP15 on affecting proteasome. A recent study has also shown that the cell surface protein, PD-L1, undergoes GSK3-dependent and β-TrCP-mediated proteasomal degradation^42^.

Targeting the TRAIL/DR5 apoptotic pathway with either recombinant TRAIL or an agonistic DR5 antibody has emerged as an attractive cancer therapeutic strategy^12, 14, 43^. Our current findings of the enhancement of DR5 activation-induced apoptosis by b-AP15 warrant further investigation of b-AP15 in combination with TRAIL or DR5 agonistic antibody as an effective cancer therapeutic regimen *in vivo* and in the clinic, and underscore the clinical translational significance of this approach. The main effector immune cell types that exert cytotoxicity against malignant cells are cytotoxic T cells and NK cells, which can generate and secrete TRAIL and kill cancer cells through TRAIL/DR5 activation-mediated apoptosis in addition to through perforin and granzyme-containing lytic granules^44^. Hence, b-AP15 may have the potential to enhance immunotherapy against cancer cells by potently sensitizing cancer cells to TRAIL-induced apoptosis. This notion is also supported by a previous study showing that b-AP15 sensitized cancer cells to killing by T and NK cells *in vitro*, which occurs in part through TRAIL-mediated apoptosis, and b-AP15 pre-treatment followed by infusion of either NK or T cells significantly delayed tumor progression in tumor-bearing mice^23^.

## Materials and Methods

### Reagents

b-AP15 was purchased from Cayman Chemical Company (Ann Arbor, MI). CFZ was purchased from Selleck Chemicals (Houston, TX). BTZ was originally provided by Millennium Pharmaceuticals (Cambridge, MA). CQ was purchased from Sigma Chemical Co. (St. Louis, MO). Baf A was purchased from LC laboratories (Woburn, MA). Human recombinant TRAIL was purchased from PeproTech, Inc. (Rocky Hill, NJ). AMG655 was supplied by Amgen, Inc (Thousand Oaks, CA). Other antibodies were the same as described previously^45^.

### Cell lines

A549, H460 and Calu-1 cells were described previously. HCT116 and its isogenic and DR5-KO cell lines were provided by Dr. Lin Zhang (University of Pittsburgh, Pittsburgh, PA) and used in our previous studies^25, 46^. A549 cells were authenticated by Genetica DNA Laboratories, Inc. (Cincinnati, OH) through analyzing short tandem repeat DNA profile. Other cell lines were not authenticated. These cell lines were grown in RPMI 1640 medium supplemented with 5% fetal bovine serum at 37 °C in a humidified atmosphere consisting of 5% CO_2_.

### Cell survival assay

Cells seeded in 96-well cell culture plates were treated on the second day with the tested agents. Cell numbers were determined using sulforhodamine B (SRB) assay as described previously^47^. The cells were seeded in 12-well plates and the next day exposed to different treatments for 60 h. The surviving cell colonies were stained with 0.2% crystal violet to determine the effect of long-term treatments on cell survival. Moreover, the stained cells were solubilized with 1% SDS for measurement of absorbance with a microplate reader at 570 nm.

### Detection of apoptosis

Apoptosis was evaluated with a Cell Death Detection ELISA^Plus^ kit (Roche Molecular Biochemicals, Indianapolis, IN) or with an annexin V/7-AAD apoptosis detection kit (BD Biosciences; San Jose, CA) according to the manufacturer’s instructions. Caspase and PARP cleavage were detected by Western blot analysis as additional indications of apoptosis.

### Western blot analysis

Whole-cell protein lysates were prepared and analyzed by Western blotting as described previously^45^.

### Detection of cell surface DR5

Cell surface DR5 expression was detected with flow cytometry as described previously^48^. The mean fluorescent intensity (MFI) that represents antigenic density on a per cell basis was used to assess cell surface DR5 levels. Phycoerythrin (PE)-conjugated mouse anti-human DR5 (DJR2-4) monoclonal antibody and PE mouse IgG1 isotype control (MOPC-21/P3) were purchased from eBioscience (San Diego, CA).

## Electronic supplementary material

Supplemental Figures
