# Supplementary material for: The proteasome deubiquitinase inhibitor b-AP15 enhances DR5 activation-induced apoptosis through stabilizing DR5
Source: Sci Rep. 2017 Aug 14;7:8027. doi: 10.1038/s41598-017-08424-w (PMC5556018; doi:10.1038/s41598-017-08424-w)
Supplement: Supplementary file 1 — Supplemental Figures [file 41598_2017_8424_MOESM1_ESM.pdf]

The proteasome deubiquitinase inhibitor b-AP15 enhances DR5 activation-induced apoptosis through stabilizing DR5

You-Take Oh, Liang Deng, Jiusheng Deng and Shi-Yong Sun*

Department of Hematology and Medical Oncology, Winship Cancer Institute, Emory University School of Medicine, Atlanta, Georgia, USA

Fig. 1A

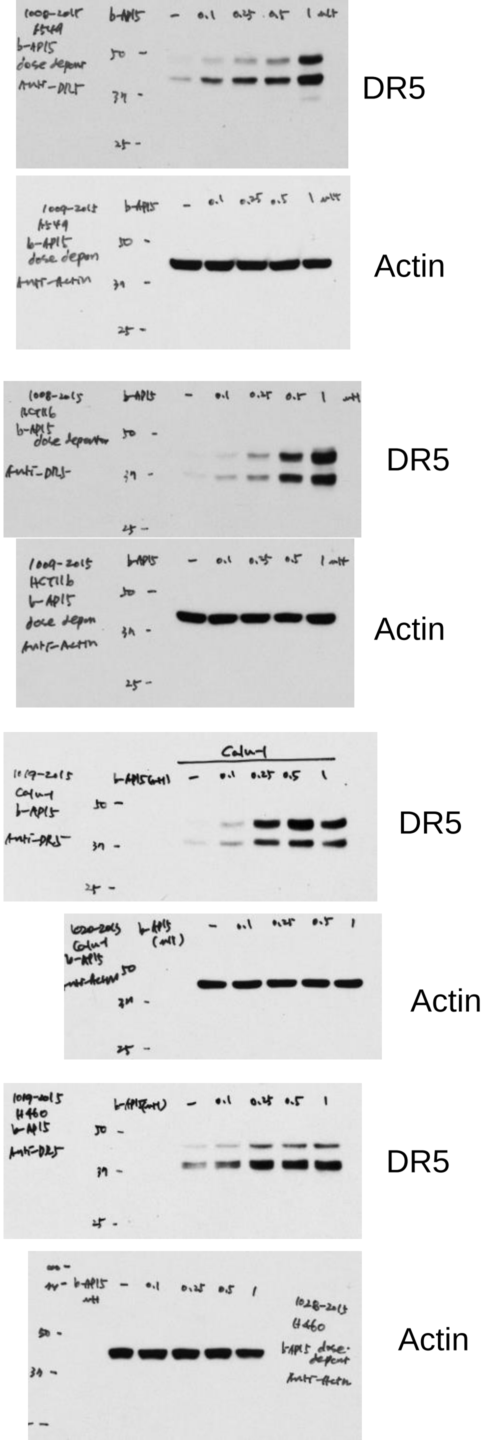

Fig. 1B

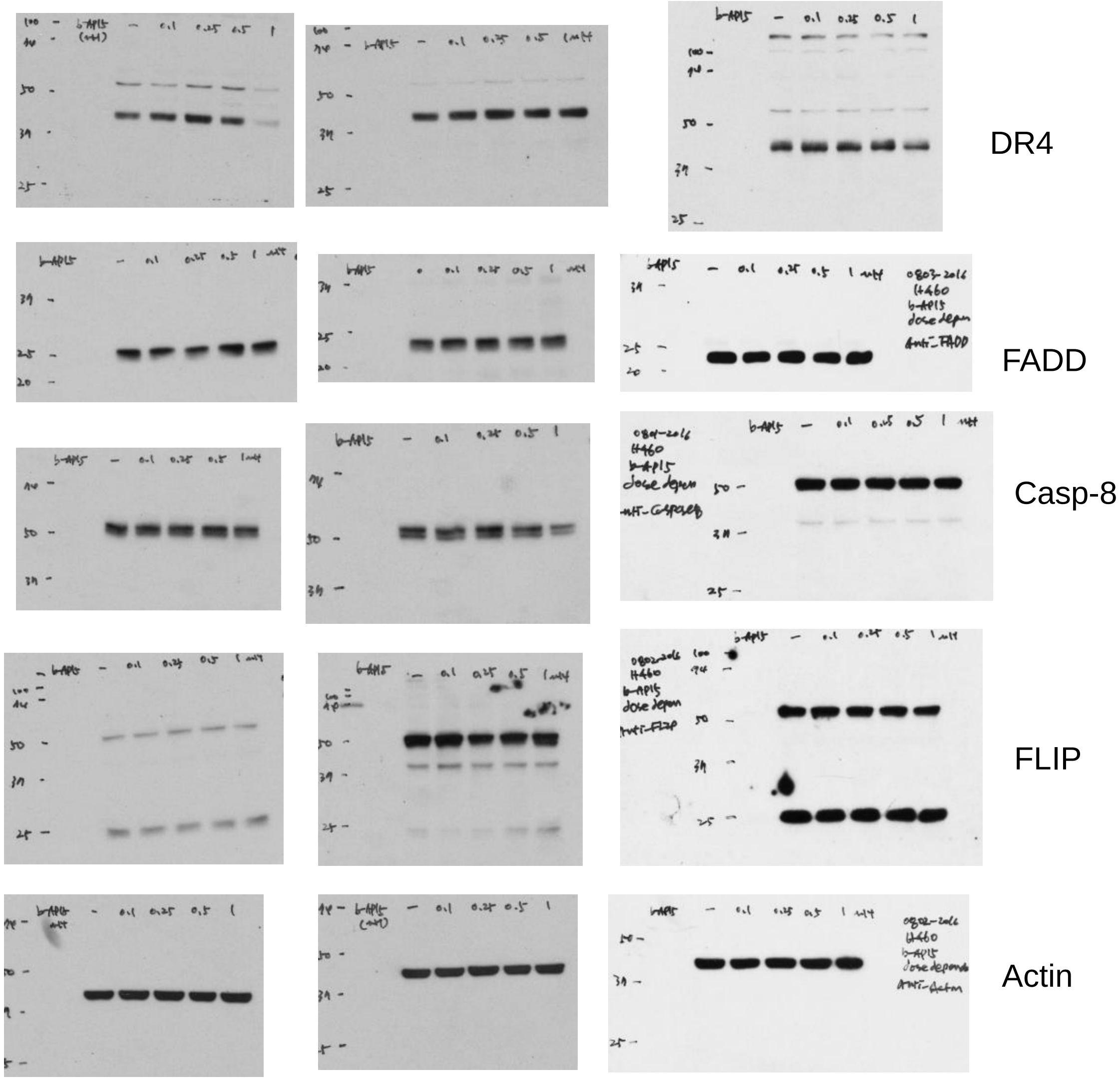

Figure S1

Fig. 2A

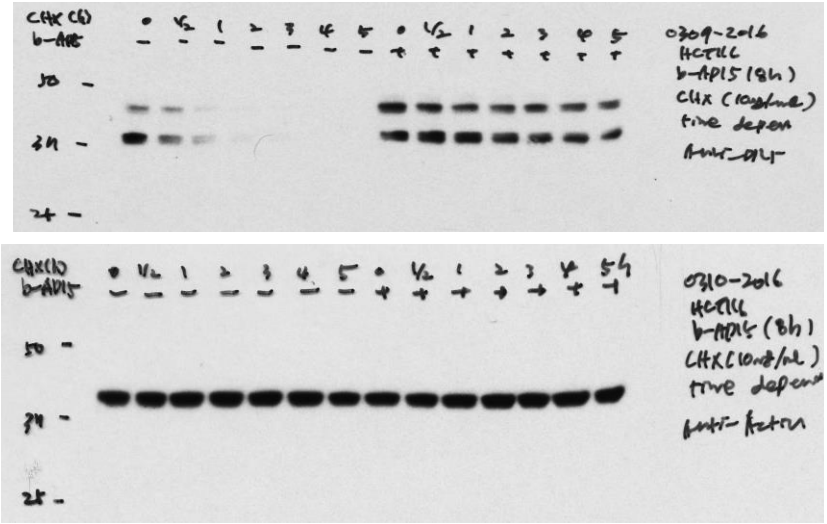

Fig. 2B

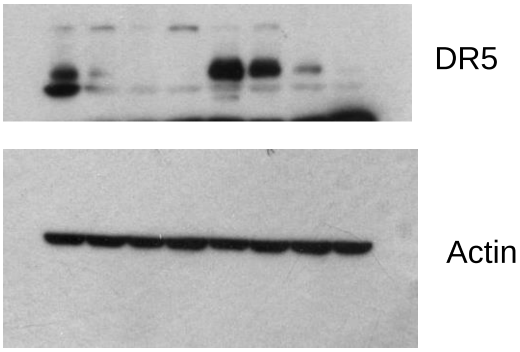

Fig. 2C

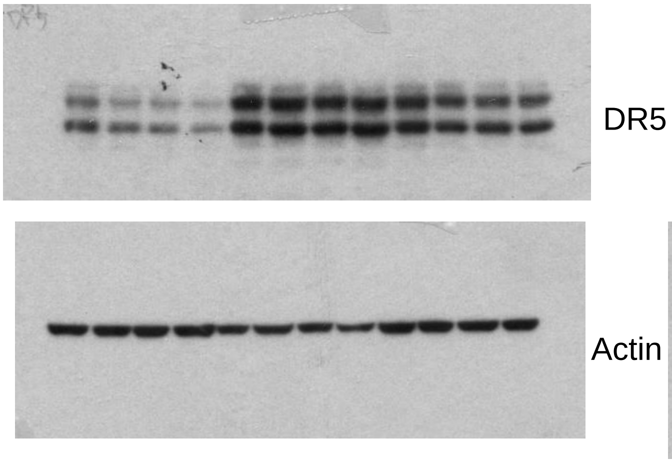

Fig. 2D

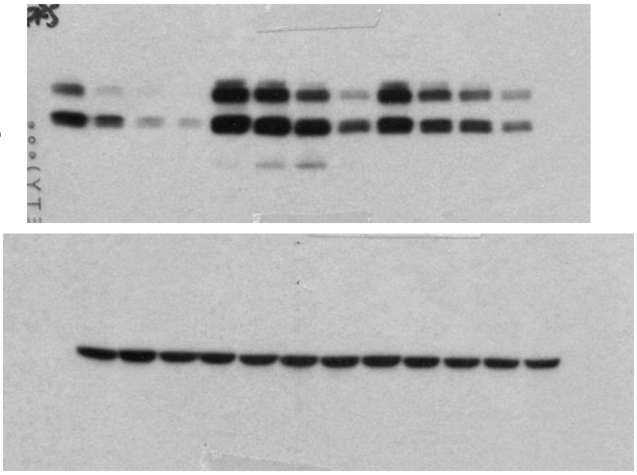

Figure S2

Fig. 3D

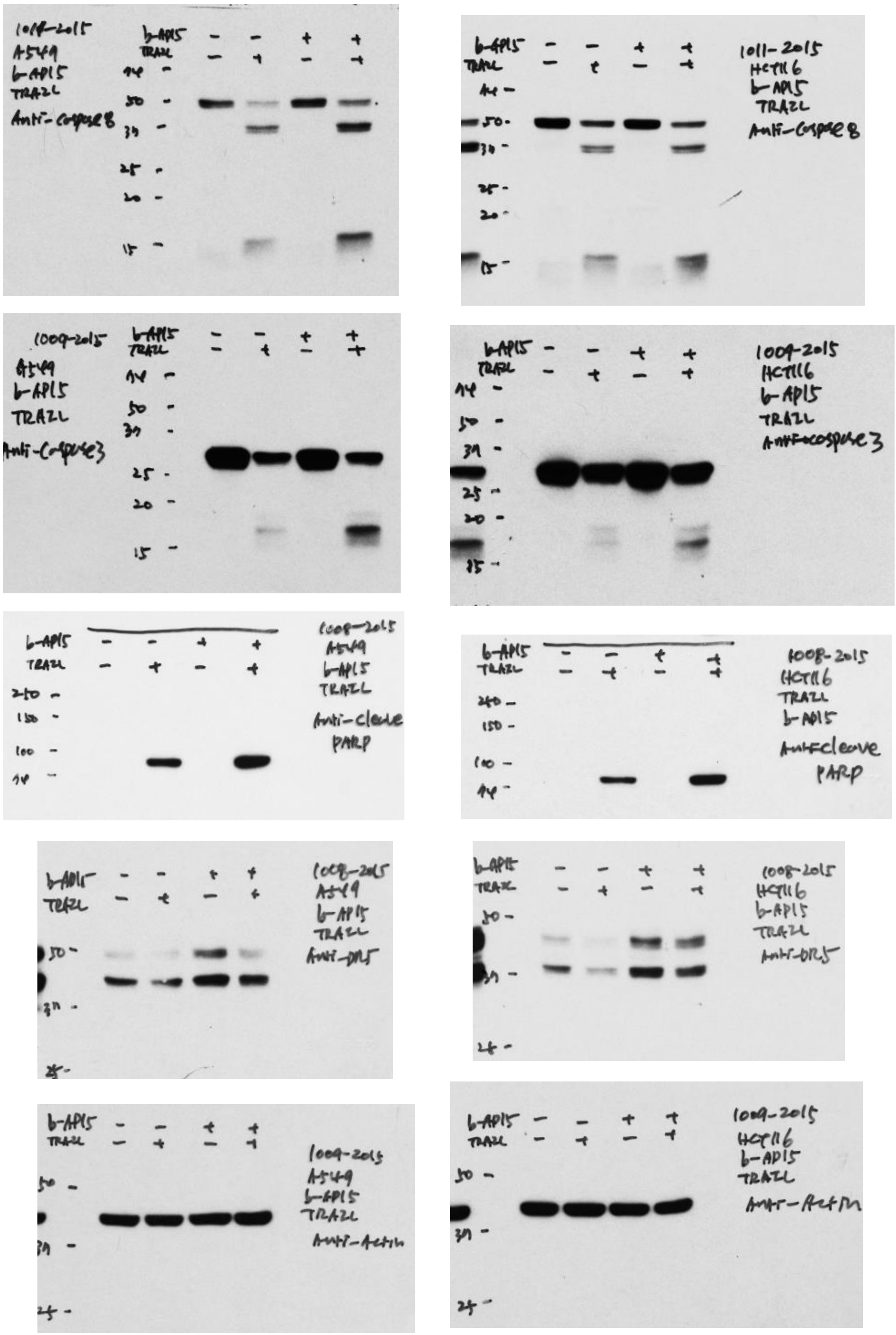

Figure S3

Fig 4D

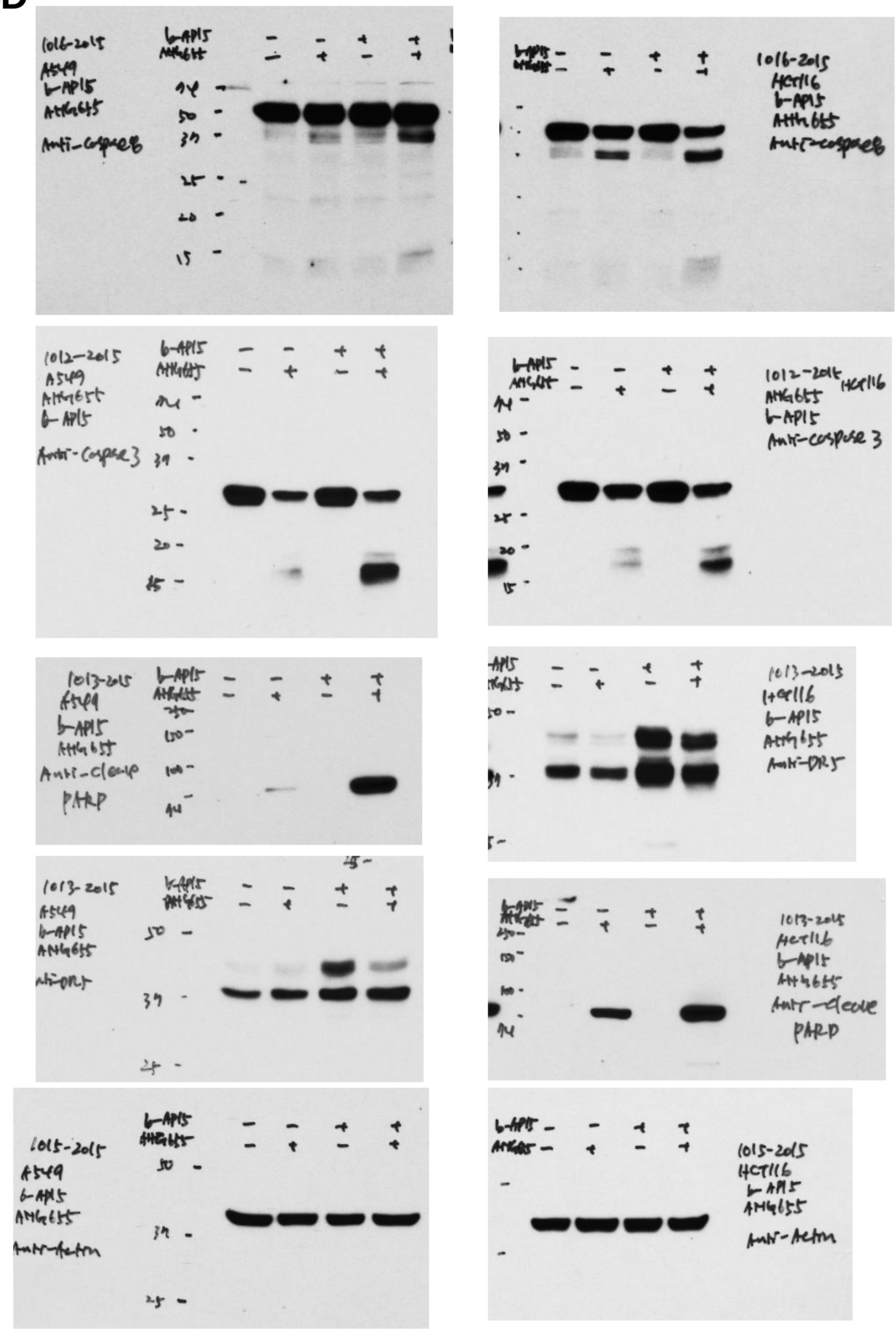

Fig 5A

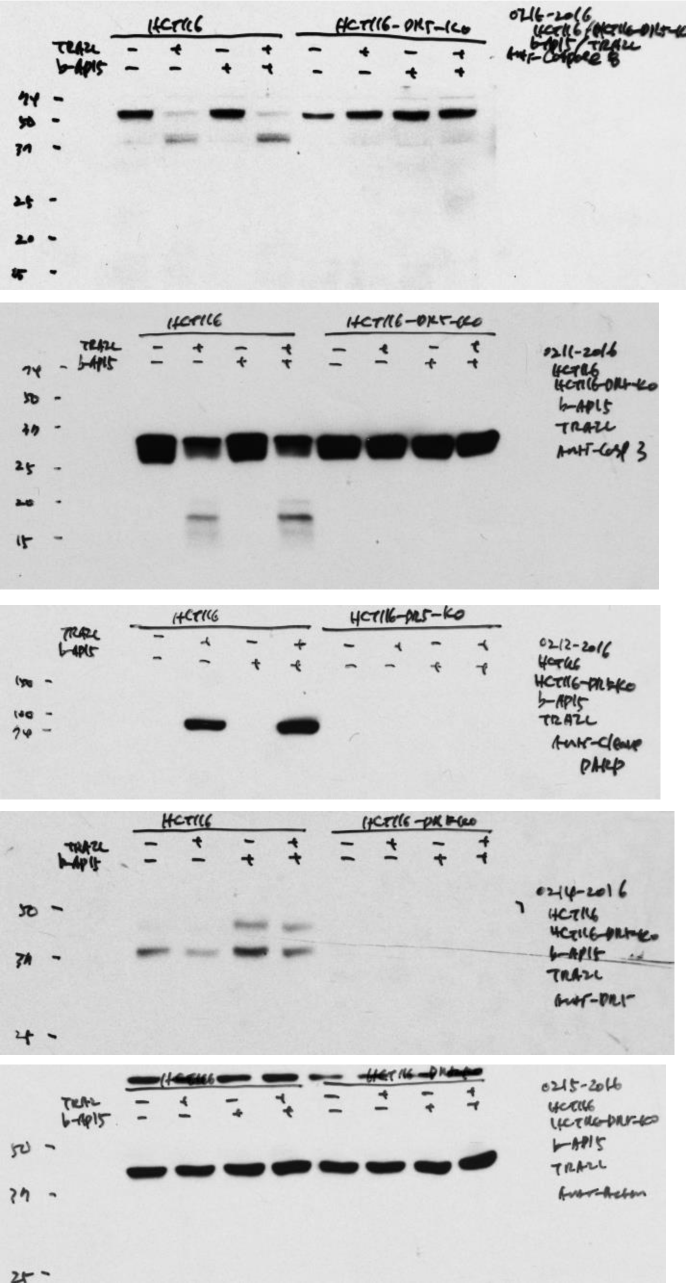

Fig 5C

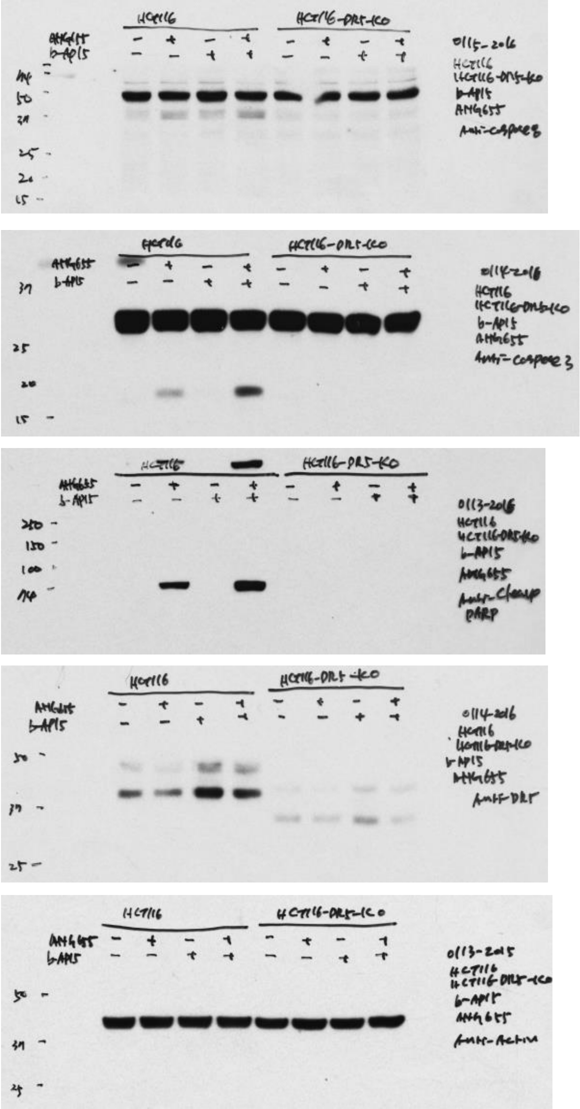

Figure S5

Fig. 6C

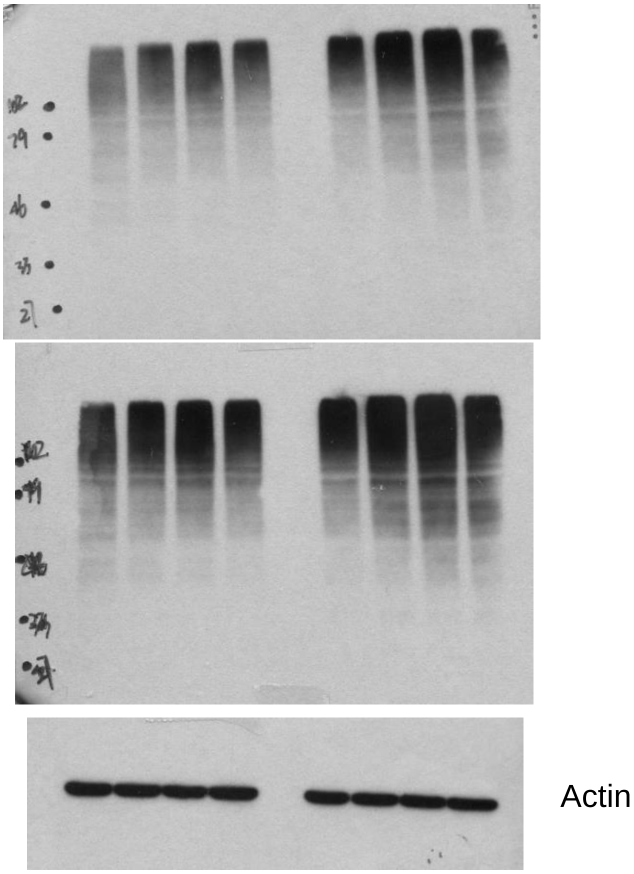

Fig. 6A

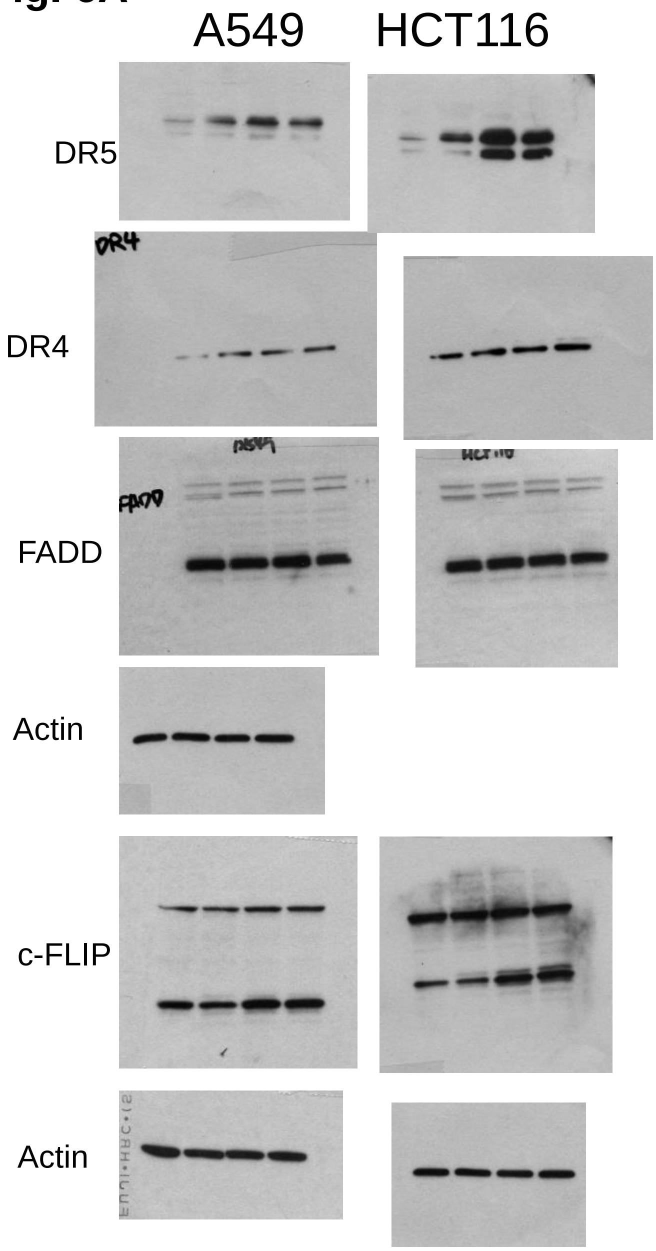

Fig. 6E

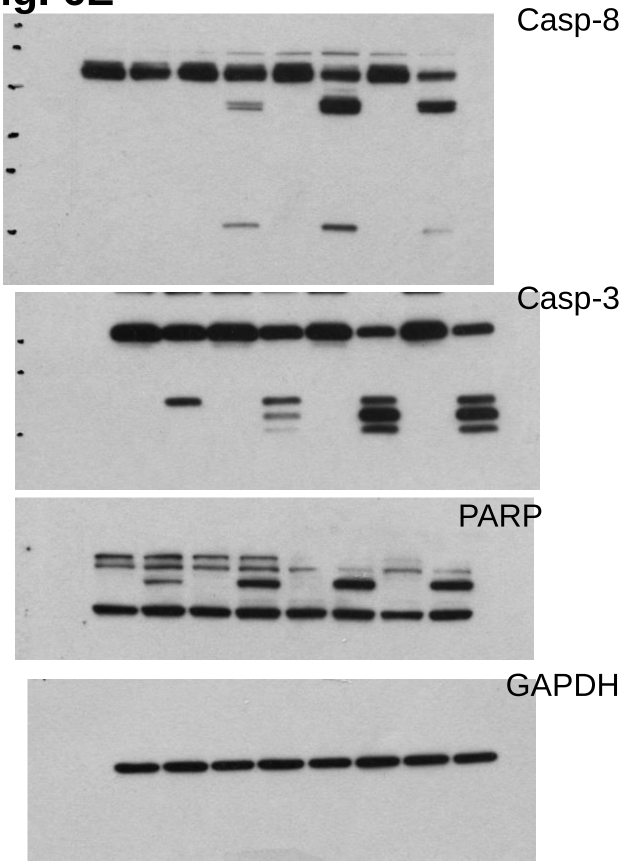

Figure S6

Fig. 7A

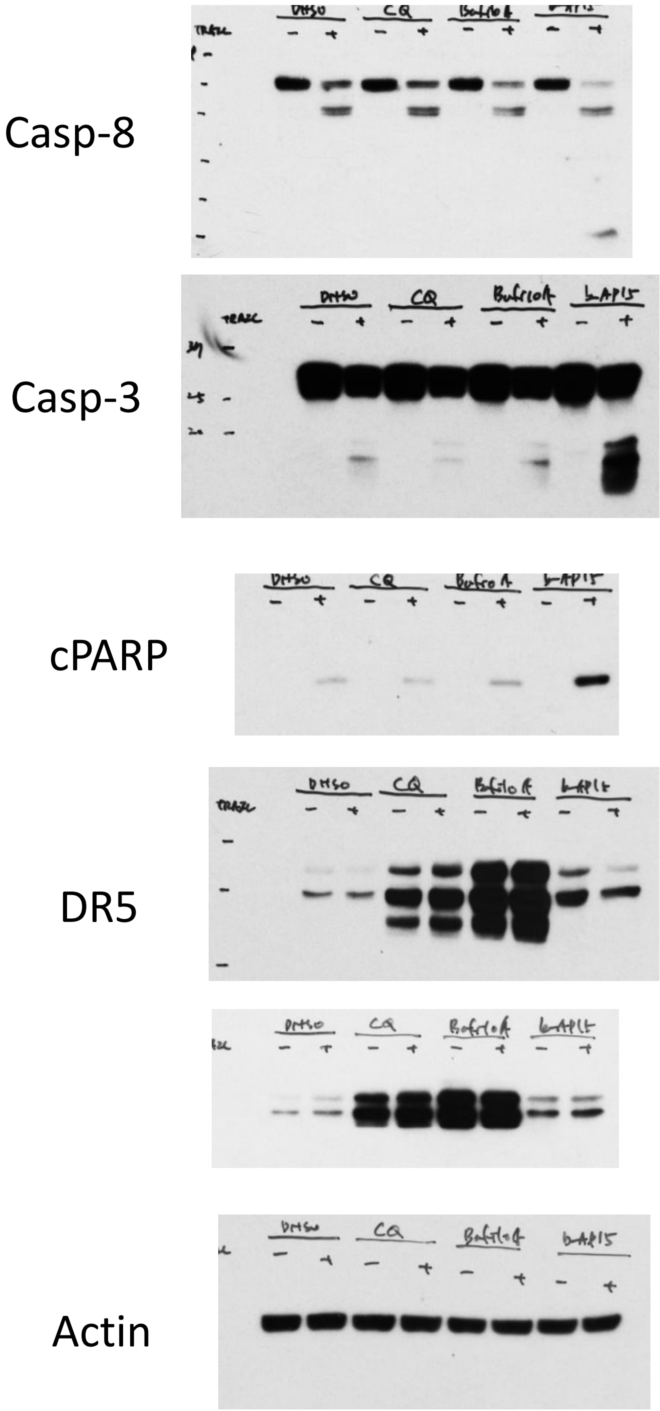

Fig. 7D

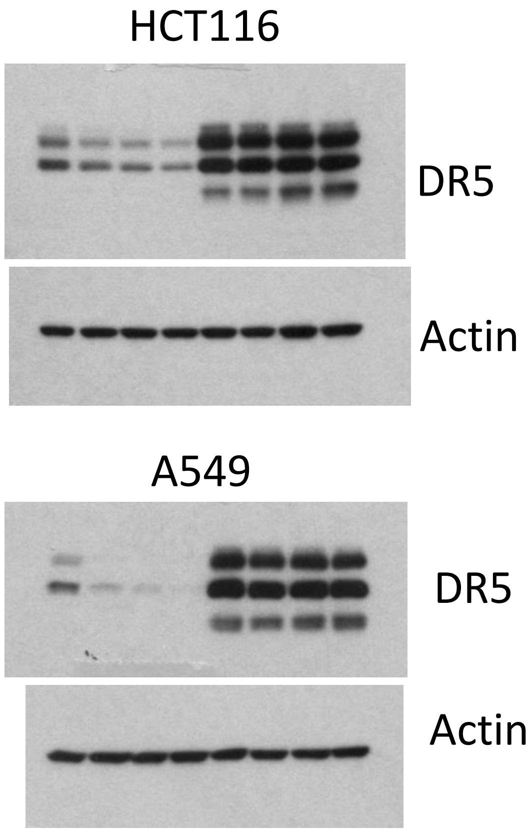

Fig. 7E

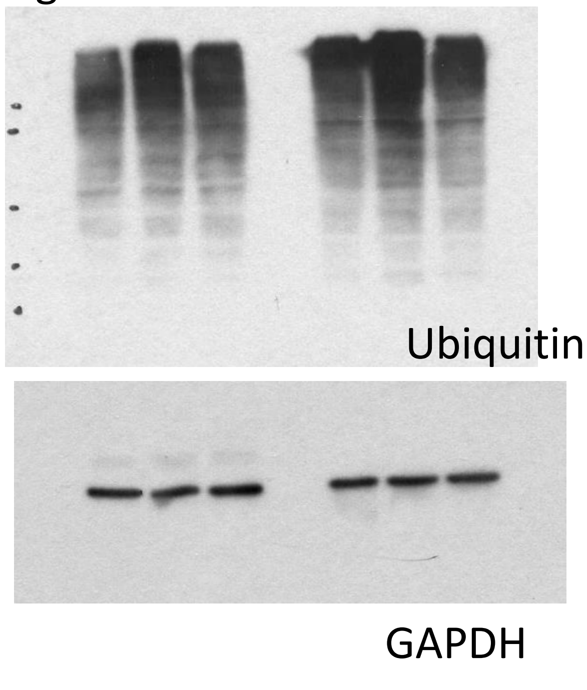

Oh et al. Fig. S7
